# South West Obstreticians and Gynaecologists Society, May 1990 Meeting

**Published:** 1990-12

**Authors:** 


### South West Obstetricians & Gynaecologists Society

### Meeting held at Plymouth Medical Centre on 18th May 1990

### ALTERNATIVES TO EXAMINATION UNDER ANAESTHESIA IN THE STAGING OF CERVICAL CARCINOMA

**James Browning MRCOG FRCS**

Lecturer in Obstetrics and Gynaecology

Bristol Maternity Hospital

EUA findings are discordant with those at staging laparotomy in 39% of cases (Averettem 1972): parametrial assessment is particularly poor.

Vaginal and rectal ultrasound offer an alternative which is well tolerated, requires no anaesthesia, does not utilise ionising radiation and is inexpensive.

Vaginal scanning in the sagittal plane images the uterine body and cervix, and determines vaginal and bladder invasion.

Transverse plane transrectal scanning allows visualisation of the cervix, its parametrium, and the rectal wall. The normal parametria image as a crescent interrupted centrally by the cervix. In benign inflammatory parametritis, a transient smooth dilatation occurs with maintenance of the cervix in the midline.

In malignant invasion there is irregular dilatation of the parametrium with shift of the cervix from the midline to the affected side.

Cervical volume may be determined by 3-plane measurement.

Parametrial cytology may be determined by fine needle aspiration under ultrasound control.

102 patients were studied.

Validation against surgical pathological speciments demonstrates a positive predictive value of 98% in the assessment of parametrial invastion.

(Magnetic Resonance Imaging performed well in parametrial assessment. It has particular value in detecting recurrence, but is costly. The use of CT scanning should be confined to examination of the large tumour and para aortic lymph nodes.).

### FETAL ECG WAVEFORM ANALYSIS IN LABOUR

**Jenny Westgate MRCOG**
Fetal Research Group, Department of Obstetrics, Plymouth General Hospital

Attempts to improve the predictability of current heart rate based intrapartum fetal monitoring have lead to renewed interest in analysis of the fetal electrocardiographic waveform obtained from a scalp electrode. The most useful features appear to be the ratio of the PR to the RR interval and the ST waveform. The Plymouth Fetal Research group has been instrumental in organising a multicentre EEC study to collect a large fetal ECG database which will be archived at Plymouth. The Research Group has assessed the standardised data collection system used to ensure the waveform is not corrupted during collection. A randomised clinical study comparing different fetal scalp electrodes has clearly shown that single spiral electrodes produce the best quality ECG signals thus are most suitable for data collection. The filter characteristics of the processor to be used have been validated and software has been developed to enable both the raw ECG and patient data to be collected onto an optical disc storage system. The relationships between the ECG variables, antenatal and intrapartum events and eventual outcome will be investigated. Expert systems technology will be used to develop a portable, intelligent fetal monitor to provide consistent, objective information on fetal condition in labour.

### THE IMPORTANCE OF MONITORING LONGTERM OESTROGEN IMPLANT USERS

**Malcolm Padwick MRCOG**
Plymouth General Hospital

Supraphysiological plasma oestradiol levels and prolonged endometrial stimulation, lasting many months after cessation of treatment, have been reported in women following repeated oestradiol implants. It has also been reported that prolonged exposure to oestrogens increases the risk of carcinoma in the postmenopausal breast.

One hundred women receiving oestrogen implants were followed for 12 months. At every visit each woman completed a "graphic rating scale" (GRS) recording severity of symptoms traditionally related to oestrogen deficiency. Venous blood was taken for asessment of plasma oestradiol and any previous implant therapy recorded. GRS scores failed to correlate with plasma oestradiol values. There was a strong positive correlation between plasma oestradiol and total number of implants received, 42% of women who had received 4 or more implants had plasma oestradiol levels between 700 and 1500 pmols/1. Higher values were more common when implants were administered more frequently and in larger doses. Plasma oestradiol levels reported in this study represent trough values. Post implant peak values would be significantly higher. If supraphysiological plasma oestradiol levels and their potential hazards are to be avoided regular monitoring is required. As traditional symptoms are an unreliable indicator of plasma oestradiol levels, it is suggested that all women receiving 4 or more implants are monitored by regular blood sampling. Attempts should be made to reduce both implant dosage and frequency in women embarking on long term therapy.

### PSYCHOLOGICAL APPROACH TO THE UNSTABLE BLADDER

**Bob Freeman MD MRCOG**
Plymouth General Hospital

The aetiology of the unstable bladder (D.I.) remains unknown. Treatment is often unsatisfactory. The effect of emotions on the lower urinary tract has been well documented. Support for a possible psychosomatic aetiology in D.I. comes from well-known clinical findings, such as provocative stimuli (e.g. sound of running water), psychological questionnaire studies, the high placebo response from drug trials and the objective improvement from "psychological" treatments.

As psychological factors are probably implicated, bladder-drill, biofeedback, hypnosis, acupuncture and psychotherapy have been tried.

Results of bladder-drill are good in the short-term but in the long-term show a high relapse rate.

Hypnosis is a form of unconscious bladder re-training. Our work showed good results initially but at two years there was also a high rate of relapse. Attempts to prevent relapse by using casette tapes were ineffective.

Similar outcomes have been noted with biofeedback and psychotherapy.

In summary, patients with an unstable bladder should have known causes e.g. a neuropathy or outlet obstruction excluded by history, examination and investigations, including Urodynamics.

Treatment involves a good Doctor/Patient relationship allied to psychotherapy, bladder-drill and drug therapy. The patient is monitored as an outpatient initially. If there is no response then in-patient treatment is indicted. If this fails, then hypnotherapy and/or biofeedback should be tried and if successful should be continued indefinitely to prevent relapse.

Only when all this has failed and the symptoms are severe does one consider surgery e.g. "Clam" ileocystoplasty.

### THE SQUID — GIANT AXON

**Dr Quentin Bone FRS**
Marine Biology Association, Plymouth

The Plymouth laboratory of the Marine Biological Association opened in 1888, as a result of the success of the Stazione Zoologica, Naples, on which the Plymouth laboratory was modelled. Plymouth was chosen as the site for the UK marine laboratory, since the fauna and flora are particularly rich. Curiously, the first Director, Mr W Heape, had gynaecological interests! The aims of the Association were to improve zoological and botanical science, and to increase knowledge of the habits and food of British food fishes and molluscs. This second applied aim was soon taken over mainly by the Lowestoft laboratory of the Ministry of AGriculture, Fisheries and Food (originally an outstation of Plymouth). Although some applied work has been done at Plymouth, notably during the Torrey canyon oil spill, its staff and visitors have mostly been concerned with fundamental researches. Perhaps the most famous work done at Plymouth began in 1938, when, A L Hodgkin and A Huxley started their classical research on the ionic basis of nerve action potentials, using the giant axon of the squid (up to 1.5 mm in diameter) discovered in the early thirties by J Z Young. Already in 1938 they had recorded intracellular nerve action potentials for the first time, and their subsequent work after the war using voltage clamp, internal perfusion, and traces to monitor ion fluxes led to the award of the Nobel Prize in Physiology in 1953. Part of a film taken at Plymouth was shown, in which J Z Young, A L Hodgkin, and other workers on squid axons at Plymouth demonstrated their techniques.

### OUT-PATIENT DIAGNOSTIC HYSTEROSCOPY

**Tony Falconer MRCOG, Peter De Jong and Frances Doel**
Plymouth General Hospital

This prospective Study was designed to evaluate out-patient hysteroscopy using para-cervical block. Over two years 328 patients underwent diagnostic hysteroscopy under local anaesthesia. A hysteroscopic examination was performed in an out-patient suite specially prepared for this procedure and thirty minutes were allocated to each examination.

A para-cervical or intra-cervical block of 4 mls Lignocaine 1% was injected via a dental syringe into the cervix. A rigid 4 millimeter diagnostic hysteroscope was passed, facilitated by carbondioxide uterine insufflator. A thorough examination and assessment of the endometrial cavity was made and if appropriate, an endometrial biopsy was taken by either Acurette or fine curette. The level of pain and discomfort experienced during the examination was matched to one of five categories, from 1 — intolerable pain, through to 5 which was easily acceptable discomfort.

320 patients were included in the Study with a medium age of 49 years. 38% of the patients were referred for post menopausal bleeding and 55% for dysfunctional uterine bleeding. The examination was successful in 314 of 328 cases. Failure was usually due to cervical stenosis or cervical canal obstruction. No abnormality was detected in 50% of the cases. Fibroid or endometrial polyps were found in 17% and suspected neoplasia 8%. In 20% atrophic endometrium was visualised. In 49 patients a second examination under general anaesthesia was performed. In 35 cases this was for surgical polypectomy. Five cases were suspected malignancy and inadequate biopsy were included, and two of these showed carcinoma of the endometrium. Two showed glandular hyperplasia and one was normal. Nine additional cases were included due to poor visualisation of the cavity and this group included one malignancy. Fifty-five percent of the histology was normal. Endometrial hyperplasia was found in 5% and endometrial malignancies in 4%. Hysteroscopic and histological findings showed good correlation. However, in three cases suspected neoplasia was not confirmed histologically.

Most women found the procedure acceptable, nd only five found it intolerable. No complications attributed to the procedure occurred, and none required admission after hysteroscopy. The side effects were mild and transient and included abdominal cramps, shoulder tip pain, and vagal attacks in two women. Two patients experienced severe discomfort, such that the procedure was abandoned.

These data suggest that out-patient diagnostic hysteroscopy under local anaesthesia is a well tolerated procedure which considerably reduces the need for hospital admission. It provides early investigation for patients with a variety of gynaecological disorders at low cost and with minimal facilities. To the best of our knowledge, no malignancy has been overlooked in any of the 328 patients undergoing this examination.

### THE USE OF RU486 IN THE INTERRUPTION OF EARLY PREGNANCY

**Jane Norman — Research Registrar**
Department for Obstetrics and Gynaecology and MRC Reproductive Biology Unit, University of Edinburgh
**R W Kelly**
**D T Baird**

Since the isolation of progesterone in 1934, studies have shown that it is indispensable for the formation of secretory endometrium and hence nidation and implantation. It also appears to maintain uterine quiescence as shown by Csapo (1973). The antiprogestin RU486 is a progesterone and glucocorticoid receptor antagonist synthesised in 1978 in the laboratories of the French company Roussel Uclaf. Studies using RU486 alone as an abortifacient have demonstrated a complete abortion rate of around 61% in women up to 56 days gestation. However RU486 also sensitizes the uterus to the action of exogenous prostaglandins, and the addition of a subtherapeutic dose of exogenous prostaglandin gives an abortion rate of 95–100%.

Ru486 increases uterine activity in vivo and also increases endometrial prostaglandin production in vitro. In studies using RU486 with or without the prostaglandin synthetase inhibitor indomethacin, we have shown that RU486 in vivo increases the ability of the endometrium to generte prostaglandins and increases uterine activity. The concurrent addition of indomethacin inhibits prostaglandin production by the endometrium but fails to inhibit the increase in uterine activity. This suggests that factors other than an increase in prostaglandin production are responsible for the increase in uterine contractility seen after RU486.

### "THE ARMADA"

**Tristram Besterman MA, FMA, FCS**
City Curator, City of Plymouth

The events leading up to the Armada from the first decades of the sixteenth century were described. Sectarian division split Christian Europe, with hideous outrages perpeterted on each other by Catholic and Protestant regimes. International tensions were fuelled by the piracy of English adventurers such as Drake against Spanish possessions and treasure ships in central and South America. Provoked beyond measure, Philip's divine mission to rid England of the Protestant 'usurper' Elizabeth, was given temporal legitimacy through his earlier marriage to Elizabeth's predecessor, Queen 'Bloody' Mary Tudor. The roles of two great Devon seadogs Drake and Hawkyns were examined to disentangle fact from fiction. The failure of the Spanish Armada to invade England resulted as much from ill luck and a fatally flawed strategy, as from the tactical brilliance of the English. Of the 30,000 who set out from Spain, only 10,000 returned — the rest perished from disease, starvation and drowning on the long journey home. The ten days of skirmishes in the English Channel were curiously inconclusive. From the failure of Philip's ill-conceived 'Enterprise of England' can be traced the slow decline of Spain's influence and the establishment of England's confidence as a maritime power, laying the foundations of the British Empire.

A single gold ring recovered in the 1960's from one of the Armada wrecks on the coast of Ireland, was a keepsake from a lover. It is inscribed 'No tengo mas que dar te': I have nothing more to give thee. A poignant memorial to all those caught up in the lunacy of international conflict.

